# *In Vivo* Systematic Analysis of *Candida albicans* Zn2-Cys6 Transcription Factors Mutants for Mice Organ Colonization

**DOI:** 10.1371/journal.pone.0026962

**Published:** 2011-10-31

**Authors:** Patrick Vandeputte, Françoise Ischer, Dominique Sanglard, Alix T. Coste

**Affiliations:** Institute of Microbiology, University of Lausanne and University Hospital Center, Lausanne, Switzerland; University of Minnesota, United States of America

## Abstract

The incidence of fungal infections in immuno-compromised patients increased considerably over the last 30 years. New treatments are therefore needed against pathogenic fungi. With *Candida albicans* as a model, study of host-fungal pathogen interactions might reveal new sources of therapies. Transcription factors (TF) are of interest since they integrate signals from the host environment and participate in an adapted microbial response. TFs of the Zn2-Cys6 class are specific to fungi and are important regulators of fungal metabolism. This work analyzed the importance of the *C. albicans* Zn2-Cys6 TF for mice kidney colonization. For this purpose, 77 Zn2-Cys6 TF mutants were screened in a systemic mice model of infection by pools of 10 mutants. We developed a simple barcoding strategy to specifically detect each mutant DNA from mice kidney by quantitative PCR. Among the 77 TF mutant strains tested, eight showed a decreased colonization including mutants for orf19.3405, orf19.255, orf19.5133, *RGT1*, *UGA3*, orf19.6182, *SEF1* and orf19.2646, and four an increased colonization including mutants for orf19.4166, *ZFU2*, orf19.1685 and *UPC2* as compared to the isogenic wild type strain. Our approach was validated by comparable results obtained with the same animal model using a single mutant and the revertant for an ORF (orf19.2646) with still unknown functions. In an attempt to identify putative involvement of such TFs in already known *C. albicans* virulence mechanisms, we determined their *in vitro* susceptibility to pH, heat and oxidative stresses, as well as ability to produce hyphae and invade agar. A poor correlation was found between *in vitro* and *in vivo* assays, thus suggesting that TFs needed for mice kidney colonization may involve still unknown mechanisms. This large-scale analysis of mice organ colonization by *C. albicans* can now be extended to other mutant libraries since our *in vivo* screening strategy can be adapted to any preexisting mutants.

## Introduction

*Candida albicans* is an ubiquitous dimorphic organism colonizing skin and mucosa of immuno-competent people without causing any pathologies. In contrast, it is the cause of a wide spectrum of diseases in immuno-compromized patients, ranging from benign mucosal infections such as oral thrush to disseminated candidiasis, which can be fatal. Oral and vaginal infections with *C. albicans* are extremely common even in weakly immuno-compromized individuals. In severe cases of immunodeficiency, *C. albicans* penetrates into deeper tissues and may enter the bloodstream. From the bloodstream, the fungus has the potential to invade almost all body sites and organs. Therefore, *C. albicans* is able to survive in radically different environments with dramatic changes in physico-chemical conditions such as oxygen and carbon dioxide tension, pH and temperature. In addition, it has to escape host immune defenses, even if they are weakened in immuno-compromized patients [1], [2].

The therapy of *C. albicans* infections necessitates the use of antifungal agents acting essentially against ergosterol, DNA and cell wall biosynthesis as described in details in several reviews [3], [4], [5], [6]. The exposure of fungal pathogens to antifungal agents can engage mechanisms enabling their adaptation and finally resulting in drug resistance that is associated with treatment failure. *C. albicans* resistance to antifungal agents is often the result of genetic alterations such as gain-of-function mutations or chromosomal rearrangements (for review see [7]), or of the formation of multicellular associations known as biofilms [8], [9]. In the case of *C. albicans* systemic infections, treatment failures are relatively frequent even in absence of *in vitro* measurable antifungal resistance in specific fungal strains, leading to a mortality rate of up to 30%. Therefore, it seems unlikely that existing antifungal agents will have an impact on the mortality rate of systemic fungal infections. A better understanding of host-pathogen interactions is necessary to develop either new drugs and/or new clinical approaches. These new therapeutic approaches may be combined with existing antifungal chemotherapies and immunotherapies [10].

Several studies have addressed this question in the past but focusing only on the host immune response involving chemokines, cytokines and effector cells [11], [12], [13], [14]. These studies often included analyses on a restricted number of genes or explored transcriptome analysis essentially by *ex vivo* experiments [15], [16], [17], [18], [19], [20] and one by *in vivo* analysis [21]. All these analyses yielded a limited perspective on the interactions between *C. albicans* and its host during the infectious process. Large scale reverse genetic analyses of *C. albicans* genes involved in environmental adaptation have been performed *in vitro* [22], and thus are not appropriate for the study on host-pathogen interactions. Very recently two studies performed *in vivo* large scale analyses of *C. albicans* genes involved in the infectious process [23], [24]. Nevertheless these analyses did not focus on a specific class of genes and were not exhaustive [23] or investigated heterozygous mutants of essential genes but in which the effect of the deletion of only one gene copy could not be visible [22]. Their implementation was time- and cost-consuming and could not be adapted to a pre-existing *C. albicans* mutant library.

In this study we propose a simple way to screen directly for host colonization a collection of *C. albicans* Zn2-Cys6 transcription factor (TF) mutants in a murine disseminated infection model. TFs integrate several signals originating from the environment and mediate adapted responses by modulating gene transcription. Blocking a TF involved in the response to a specific host condition may disarm the adapted fungus response that otherwise allows its survival. Previous contributions confirm this hypothesis and demonstrate that in *C. albicans*, deletion of genes encoding for TFs result in significant decrease of virulence. These TFs are involved in (i) adaptation to oxidative stress (*CAP1* and *RIM101*) [25], [26], [27], [28], (ii) nitrogen regulation (*GAT1*), and (iii) yeast to hyphae transition (*CPH1*, *EFG1*, *TUP1, and CaNDT80*) [10], [29], [30], [31], [32], [33]. Thus, TFs represent interesting candidates to study virulence in order to design new antifungal strategies. Among existing *C. albicans* TFs, the Zn2-Cys6 “zinc cluster” subclass is of particular interest since it is fungal-specific. In order to address the involvement of TF of this family in host colonization, we developed a system in which *C. albicans* TF mutants are analysed in pools of 10 tagged strains. After *C. albicans* DNA extraction from mice organs, quantitative PCR (qPCR) is performed on specific tags and thus allows the relative quantification of individual mutant in the population in infected tissues. We tested 77 mutants of specific Zn2-Cys6 TF for their potential in colonizing mice kidneys in a mouse model of systemic infection.

We were able to distinguish 8 and 4 mutants with reduced and increased colonization, respectively, as compared to an isogenic wild type strain. In an attempt to identify the putative function of these TFs and to better elucidate their involvement in virulence and/or colonization, we determined *in vitro* susceptibilities to heat, pH and oxidative stresses as well as hyphae formation and agar invasion of these 77 strains. These phenotypic tests revealed an overall poor correlation with *in vivo* results, suggesting that most of these TFs may regulate the expression of yet unidentified virulence factors.

## Materials and Methods

### Strains and media

The *C. albicans* strains used in this study are listed in Supplementary File S1. These mutants are part of a larger collection of transcription factor mutants available at the Fungal Genetic Stock Center (http://www.fgsc.net/). They were selected for the presence of a fungal Zn2-Cys6 binuclear cluster DNA-binding domain (Pfam accession number PF00172, http://pfam.sanger.ac.uk/family/PF00172). The mutants were constructed by four different gene inactivation strategies (see Supplementary File S1), using two different parent strains, CAF4-2 [34] and BWP17 [35], which were both used as control strains throughout this study. Each TF mutant was transformed with a plasmid containing a barcode and was then renamed BCYi for “BarCoded Yeast” number i (see Table 1 and Supplementary File S1). Isolates were grown in complete medium YEPD (1% Bacto peptone, Difco Laboratories, Basel, Switzerland), 0.5% Yeast extract (Difco) and 2% glucose (Fluka, Buchs, Switzerland) or in minimal medium YNB (Yeast Nitrogen Base) (Difco) and 2% glucose (Fluka). When grown on solid media, 2% agar (Difco) was added. *Escherichia coli* DH5α was used as a host for plasmid constructions and propagation. DH5α was grown in LB (Luria-Bertani broth) or LB plates, supplemented with ampicillin (0.1 mg/ml) when required.

**Table 1:** Composition of the strain pools used for mice infection in this study.

barcode	Pool 1 = CAF4-2 background			Pools 2 to 5 = BWP17 background											
	Pool1			Pool2			Pool3			Pool4			Pool5		
	Parental strain	BCY number*	Orf mutated	Parental strain	BCY number*	Orf mutated	Parental strain	BCY number*	Orf mutated	Parental strain	BCY number*	Orf mutated	Parental strain	BCY number*	Orf mutated
STM11	DSY1691	1	orf19.7374	-	-	-	DSY3365	17	orf19.7319	DSY3418-1	48	orf19.4288	HZY29	66	orf19.3012
STM20	DSY1762	3	orf19.166	DSY3429-1	34	orf19.1035	DSY3411-2	19	orf19.2423	DSY3419-1	50	orf19.4450	HZY44	68	orf19.6203
STM43	DSY28927	5	orf19.2647	DSY3436-5	36	orf19.3405	DSY3412-2	21	orf19.255	DSY3420-1	52	orf19.5924	HZY59	70	orf19.6038
STM209	ACY176	15	orf19.3188	DSY3437-5	38	orf19.7372	HZY34	164	orf19.5133	DSY3421-1	54	orf19.5940	HZY60	72	orf19.5849
STM219	DSY3297	7	orf19.1499	DSY3447-11	40	orf19.1718	DSY3414-1	23	orf19.3252	DSY3424-6	56	orf19.2808	HZY61	74	orf19.4649
STM224	DSY3298	9	orf19.6817	HZY23	42	orf19.6985	DSY3415-1	25	orf19.4145	DSY3425-9	58	orf19.4524	HZY63	76	orf19.2623
STM227	PVY140	166	orf19.1168	HZY24	44	orf19.7371	DSY3416-1	27	orf19.4166	DSY3426-2	60	orf19.2748	HZY67	78	orf19.3190
STM232	-	-	-	HZY28	46	orf19.4766	DSY3417-1	29	orf19.4225	DSY3427-3	62	orf19.3876	DSY4160	80	orf19.7381
STM6	CAF4-2	11	WT	BWP17	31	WT	BWP17	31	WT	BWP17	31	WT	BWP17	31	WT
STM240	DSY2101	13	*CMP1*	DSY4343	82	*CMP1*	DSY4343	82	*CMP1*	DSY4343	82	*CMP1*	DSY4343	82	*CMP1*

barcode	Pools 6 to 10 = BWP17 background														
	Pool6			Pool7			Pool8			Pool9			Pool10		
	Parental strain	BCY number*	Orf mutated	Parental strain	BCY number*	Orf mutated	Parental strain	BCY number*	Orf mutated	Parental strain	BCY number*	Orf mutated	Parental strain	BCY number*	Orf mutated
STM11	DSY4178-A	84	orf19.5251	CJN411	100	orf19.3986	CJN518	116	orf19.4568	CJN592	132	orf19.1255	CJN983	148	orf19.3753
STM20	DSY4179-A	86	orf19.5097	CJN419	102	orf19.668	CJN528	118	orf19.5548	CJN593	134	orf19.3305	CJN997	150	orf19.6781
STM43	DSY4180	88	orf19.2747	CJN491	104	orf19.7518	CJN531	120	orf19.3308	CJN598	136	orf19.4573	IS08-7	152	orf19.2646
STM209	-	-	-	CJN494	106	orf19.2745	CJN548	122	orf19.6182	CJN799	138	orf19.2280	RLS8	154	orf19.3876
STM219	CMY6-1	92	orf19.5992	CJN495	108	orf19.7583	CJN563	124	orf19.431	CJN801	140	orf19.5338	RLS11	156	orf19.4998
STM224	GKO316	94	orf19.3012	CJN506	110	orf19.5729	CJN571	126	orf19.7318	CJN803	142	orf19.4767	RLS17	158	orf19.5133
STM227	I093-5	96	orf19.6888	CJN511	112	orf19.757	CJN577	128	orf19.538	CJN805	144	orf19.4776	JMR049	160	orf19.1685
STM232	CJN403	98	orf19.3187	CJN517	114	orf19.3127	CJN582	130	orf19.1497	CJN807	146	orf19.4778	JMR038	162	orf19.391
STM6	BWP17	31	WT	BWP17	31	WT	BWP17	31	WT	BWP17	31	WT	BWP17	31	WT
STM240	DSY4343	82	*CMP1*	DSY4343	82	*CMP1*	DSY4343	82	*CMP1*	DSY4343	82	*CMP1*	DSY4343	82	*CMP1*

Highlighted in grey are ORF mutated in two separated strains.

*Barcoded yeast number.

### Yeast transformation

Yeast transformation was performed as described previously [36].

### Phenotypic tests

Eight different *in vitro* phenotypes, putatively relevant for virulence and/or colonization in the host, were assessed for each of our mutants by comparison with their wild type parental strains. These phenotypic tests were performed by serial dilutions of fungal cultures onto solid agar YEPD-based plates. Yeast cultures were grown overnight in liquid YEPD and diluted to a density of 1.5×10^5^ cells/ml. Two serial 10-fold dilutions were performed to a final dilution containing 1.5×10^3^ cells/ml. Four microliters of each dilution were spotted onto YEPD-based plates and incubated for 24 to 72 h depending on the condition tested. Following this large scale screening, a refined screen was performed for mutants with a phenotype differing from the wild type. For that purpose, the same conditions were used except that six serially 5-fold dilutions were spotted on agar plates. First, heat susceptibility was tested by incubation at 42°C as compared to 35°C which corresponds to the reference condition. We also determined the susceptibility of our mutants to alkaline pH (pH 8.3 was achieved by adding 50 mM of HEPES pH 8.5 and 25 mM NaOH) or acidic pH (pH 3.35 was achieved by adding 50 mM of HEPES pH 1.9). Third, we assessed the susceptibility to 1 mM and 5 mM H_2_O_2_. Lastly, filamentation phenotype was determined by, either plating each mutant onto YEPD agar plates supplemented with 10% of fetal calf serum (FCS), growing cells in 10% FCS liquid YEPD medium for 4 h, and determining agar invasion capacity after 48 h of growth in YEPD agar plates. All plates were recorded for phenotypes after 24 and 48 h of incubation except for FCS-supplemented plates, which were analyzed after 72 h of incubation. For cells grown in liquid medium, pictures were taken using an Axiovert200 Zeiss inverted microscope with a 200× magnification. This screening was repeated twice from independent overnight cultures to assess the reproducibility of the results.

### Construction of plasmids

Tagged-plasmids were constructed from CIp30 [37] and were carrying STM tags consisting in random 40-nucleotides sequences used in *C. neoformans* [38], [39]. We chose 10 distinct STM tags with numbers 6, 11, 20, 43, 209, 219, 224, 227, 232 and 240 (see Table 2 for nucleotide sequences). First, complementary oligomers (Table 2) containing the 40-mer sequence of the STM tag flanked by the cohesive ends with NotI and DraIII restriction sites were hybridized and digested by DraIII and NotI. Then, the double-stranded oligomers were cloned into pKS(+) (Stratagene) at DraIII and NotI sites and re-cloned into CIp30 at ScaI and NotI sites. This cloning step was necessary since we discovered after sequence analysis of CIp30 near the NotI site that the published CIp30 map was not designed from a pKS(+) backbone but from a pKS(−) backbone. Therefore we had to subclone the double-stranded oligomers in a pKS(+) backbone to avoid the problem of the non-palindromic DraIII site. Finally 10 plasmids were generated from the CIp30 backbone containing each a specific tag and were called CIp30-STM6, −11, −20, −43, −209, −219, −224, −227, −232 and −240.

**Table 2:** Sequences of oligonucleotides used in this study.

Primer name	Sequence
**Primers for STM tag**	
STM6-F	gtgccatagctaccacacgatagctccccctagccccctacacgc
STM6-R	ggccgcgtgtagggggctagggggagctatcgtgtggtagctatggcacgta
STM11-F	gtgcacacgaaataaacctctaaacctcccccacaccaccccggc
STM11-R	ggccgccggggtggtgtgggggaggtttagaggtttatttcgtgtgcacgta
STM20-F	gtgatcaacaactaccgaccacgatcgacatccaacgccccatgc
STM20-R	ggccgcatggggcgttggatgtcgatcgtggtcggtagttgttgatcacgta
STM43-F	gtgccataaaaaaactagacccagctaccaatcacgctaccatgc
STM43-R	ggccgcatggtagcgtgattggtagctgggtctagtttttttatggcacgta
STM209-F	gtgctatcactctagcaatagcacaatctcgctctacccataagc
STM209-R	ggccgcttatgggtagagcgagattgtgctattgctagagtgatagcacgta
STM219-F	gtgccctaaaaccctacagcaatcacgatataccgctcccgacgc
STM219-R	ggccgcgtcgggagcggtatatcgtgattgctgtagggttttagggcacgta
STM224-F	gtgggtggtttgtgtgggtagagcgggggattgggggttaggggc
STM224-R	ggccgcccctaacccccaatcccccgctctacccacacaaaccacccacgta
STM227-F	gtgtggtcgcgggagatcgtggtttagagggagcgcggtctatgc
STM227-R	ggccgcatagaccgcgctccctctaaaccacgatctcccgcgaccacacgta
STM232-F	gtgatggcgttgtcgatatggttaggtagcgaggtcggttgcggc
STM232-R	ggccgccgcaaccgacctcgctacctaaccatatcgacaacgccatcacgta
STM240-F	gtgggcgctcgatcgcgggtgggtgttggatcggggtggatgggc
STM240-R	ggccgcccatccaccccgatccaacacccacccgcgatcgagcgcccacgta
**Primers for qPCR detection**	
Forward-primer	ccgtctatcagggcgatg
Primer-STM6-rev	cgcgtgtagggggcta
Primer-STM11-rev	cgccggggtggtgt
Primer-STM20-rev	catggggcgttggatg
Primer-STM43-rev	gccgcggccgcatggtagcg
Primer-STM209-rev	cggccgcttatgggta
Primer-STM219-rev	cggccgcgtcgggagcggta
Primer-STM224-rev	gcccctaacccccaatc
Primer-STM227-rev	cgcggccgcatagac
Primer-STM232-rev	gccgcaaccgacctc
Primer-STM240-rev	tcgggatctaggcttgg
Primer-STM240-for	tacgtgggcgctcgat
**Probes for qPCR detection**	
probeLNA-STM6*	[6FAM]tac+Cac+Acg+Ata+Gctcc[TAM]
probeDNA-STM11	[6FAM]CACACGAAATAAACCTCTAAACCTCCC[TAM]
probeLNA-STM20*	[6FAM]caa+Caa+Cta+Ccg+Accacga[TAM]
probeLNA-STM43*	[6FAM]aa+Cta+Gac+Cca+Gctacc[TAM]
probeDNA-STM209	[6FAM]ATCACTCTAGCAATAGCACAATCTCGC[TAM]
probeLNA-STM219*	[6FAM]ccc+Tac+Agc+Aat+Cacga[TAM]
probeDNA-STM224	[6FAM]GGTGGTTTGTGTGGGTAGAGCGGG[TAM]
probeDNA-STM227	[6FAM]TCGCGGGAGATCGTGGTTTAGAGG[TAM]
probeDNA-STM232	[6FAM]ATGGCGTTGTCGATATGGTTAGGTA[TAM]
probeLNA-STM240*	[6FAM]catccaccc+Cgatccaac[TAM]

*: For STM6, STM20, STM43, STM219 and STM240, Locked Nucleic Acid (LNA) were used as indicated (+) in the oligonucleotide sequence in order to reach a Tm suitable for qPCR reactions.

For the construction of the orf19.2646 revertant strains, pAC249 was designed to re-introduce a wild-type orf19.2646 allele at its genomic locus. For this purpose, the *SAT1* gene was amplified from pSFS2 using the primers Sat1-Not (5′-ATAAGAATGCGGCCGCGTCAAAACTAGAGAATAATAAAG-3′) and Sat1-BamHI (5′-GCAAAGGATCCCACCACCTTTGATTGTAAAT-3′). This fragment was introduced into pBluescript KS+ by BamHI and NotI to yield pDS1551. Next, orf19.2646 was amplified using the primers orf19.2646-5-for (5′-CGCGAGGTACCTCAATCAAGCCTCCTGTACC-3′) and orf19.2646-Rxho (5′-CGCGACTCGAGTGTACACAAAACTTAGAACC-3′). The resulting PCR fragment was cloned into pDS1551 by KpnI and XhoI to yield pAC249.

### Construction of orf19.2646 revertant and control strains

The plasmid pAC249 was introduced into the STM-tagged orf19.2646 mutant strain (BCY152) by SpeI digestion allowing integration at the orf19.2646 genomic locus. In parallel, the parental plasmid pDS1551 was introduced into BCY152 and into the tagged-BWP17 (BCY31) strains at the *ACT1* locus by MunI digestion.

### Animal experiments and Ethics Statement

All animal experiment were performed at the University of Lausanne and at the University hospital center under the surveillance and with the approval of the institutional Animal Use Committee: Affaires Vétérinaires du Canton de Vaud, Switzerland; with the authorization n° 1734.2 and 1734.3, according to the decree 18 of the federal law on animal protection.

Strains were grown in individual tubes for 16 hours under agitation at 30°C in YNB. Each strain was then diluted 100-fold times in YEPD and grown overnight under agitation at 30°C. Overnight cultures were washed twice in PBS and resuspended in 5 ml PBS. Concentration of each culture was measured and each strain was diluted in PBS at a concentration of 2×10^6^ cells/ml. For injection of the 10 strains pools, one ml of each diluted strain was mixed. Aliquots of each single culture and of the mix were plated onto YEDP in order to count colony-forming units (CFU).

Female BALB/c mice (20 to 25 g; Charles River France) were housed in ventilated cages with free access to food and water. To establish *C. albicans* infection, mice were injected into their lateral tail vein with 250 µl of saline suspensions containing 5×10^5^ CFU of *C. albicans* mixed strains. Groups of three mice were established for each pool or single strain infection. Group of four to five mice were used for orf19.2646 revertant experiments. Mice were monitored daily and were sacrificed three days after infection by CO_2_ inhalation. Kidneys were excised aseptically and weighted. The two kidneys were homogenized together in 5 ml sterile water and 100 µl of organ homogenates were used to count CFUs. The remaining homogenates were centrifuged and pellets were frozen overnight for further analyses. To analyse the CFU results, 10-fold dilutions of kidney extracts were plated onto YEPD supplemented with 50 µg/ml of chloramphenicol. Statistical significance of single infections was determined with the Kruskal-Wallis test.

### Preparation of *C. albicans* DNA from mice kidneys

Frozen pellets of kidneys homogenates were thawed slowly at room temperature, resuspended in 2 ml of water and then dispatched equally in 2 ml screw caps tubes. Tubes were centrifuged for 1 min at maximum speed. One pellet was kept for further analyses. The other pellets were washed three times in lysis buffer (2% Triton X-100, 1% SDS, 100 mM NaCl, 10 mM Tris HCl pH 8, 1 mM EDTA) in order to eliminate mice tissue debris. Yeast cells were resuspended in 200 µl of lysis buffer supplemented with one volume of phenol-chloroform. Cells were next disrupted by adding one volume of glass beads and by agitating the solution for 15 seconds at a power of 5 in a Fastprep 24 instrument (MP Biomedical, France). Suspensions were then centrifuged for 10 min at maximum speed. The aqueous phase was recovered and DNA was precipitated with 2 volumes of absolute ethanol. Pellet was resuspended in 50 µl of TE (10 mM Tris HCl pH 7.5, 1 mM EDTA) in which 10 µl of RNase A (Roche) (10 mg/ml) was added. Solution was incubated for 30 min at 37°C. Ten µl of proteinase K (Sigma) (10 mg/ml) were added and solution was incubated for 30 min at 65°C. DNA was then precipitated and resuspended in TE. After overnight incubation at 4°C, the concentration of DNA was measured using a Nanodrop ND1000 equipment (ThermoScientific, DE, USA).

### Quantitative real-time PCR (qPCR) and normalization

qPCR reactions were performed with 0.2 µM of each primer, 0.1 µM of probe and iTaq supermix with ROX (BioRad, Reinach, Switzerland) according to the manufacturer's instructions. Cycling conditions were as following: 2 min at 50°C, 3 min at 95°C followed by 40 cycles of 15 s at 95°C and 1 min at 60°C. Amplification and detection of PCR products were performed with a Step One Plus™ (Applied Biosystems, CA, USA). Reactions were performed in a total volume of 25 µl. Data were analysed using the Step One software V2.1 (Applied Biosystems, CA, USA). In each run, a PCR control was performed with yeast DNA extract from kidneys of non-infected mice. The signals obtained gave the noise level of the qPCR. Each sample showing a C_T_ for STM6 (wild type strain) higher than that of the non-infected mice reaction was rejected since *C. albicans* DNA extraction from kidneys probably failed. Such samples were eliminated for further analysis. Standard curves were created with 10-fold serial dilutions (10^5^ to 10^1^ copies) of the ten CIp30-STM plasmids. These ten standard curves (one for each barcode) were used to determine the copy number (Qx) of the different barcodes carried by strains of each pool from infected mice kidney (Qx_INVIVO_) or from 24h YEPD cultures (Qx_INVITRO_). To determine an increase or decrease of growth of strains *in vivo* as compared to *in vitro* conditions, the “*in vivo/in vitro”* ratio (dQx =  Qx_INVIVO/_Qx_INVITRO_) was calculated for each STM barcoded strain. This dQx ratio calculation allows the normalization of the *in vivo* data to the *in vitro* growth of each mutant strain. Finally, to obtain the colonization score of each strain (S  =  log2 (dQx/dQ_STM6_)), each dQx ratio was normalized to the dQ_STM6_ of its pool in order to normalize the results of all pools. The STM6 barcode was contained in isogenic wild type strains present in all the pools as positive control.

## Results

In order to discover new *C. albicans* virulence factors and to help elucidating novel elements in host-pathogen interactions, we propose here to screen a collection of *C. albicans* transcription factor mutants in a murine disseminated infection model.

**Figure 1:**
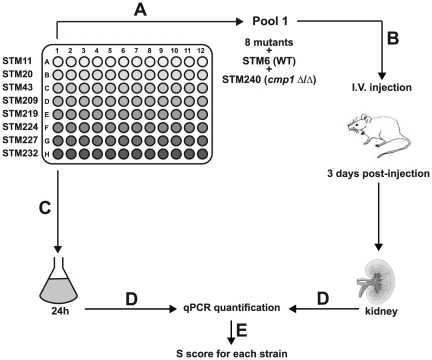
Flow chart describing the principal steps (A to E) of *in vivo* screening of the TF mutant collection. **A:** To enable the simultaneous testing of several mutants in animals, strains are tagged with 40 nucleotides barcodes. **B:** Mice are infected with pools of 10 barcoded strains. **C:** In parallel, pools are grown 24-hours *in vitro*. **D:** *C. albicans* DNA are extracted from mice kidneys and *in vitro* co-culture. **E:** Each barcoded mutant of the pool is relatively quantified by real-time qPCR in DNA extracted from mice organs and *in vitro* co-cultures.

**Figure 2:**
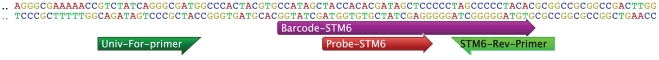
CIp30-STM6 detection. Details of the CIp30 plasmid sequence, in which the barcode (here STM6) was cloned between DraIII and NotI restriction sites. The localization of the STM6 barcode (**purple**), the position where the Taqman probe specific for STM6 is hybridizing (**red**) and the positions of forward (**dark green**) and reverse primers (**light green**) are indicated. Note that the reverse primer hybridizes at the 3′-extremity of the barcode and thus enhances the specificity of detection.

The murine disseminated infection model is a conventional experimental model to study *C. albicans* pathogenicity. In this model, several organs such as brain, spleen, lungs, liver and kidneys are infected by *C. albicans*. We planned to use this model of infection to evaluate organ colonization by Zn2-Cys6 TF mutant strains. For this purpose, we used mutants from an already existing collection containing 239 mutants corresponding to almost all the mutants of non-essential TF genes of *C. albicans* [40], [41], [42]. To reduce the number of animals used in this study, the 77 Zn2-Cys6 TF mutant strains were tested by pools as described in Figure 1 and Table 1. We developed a strategy to tag strains of a pool with 40-mers barcodes enabling their specific and individual detection (see Material and Methods and Figure 2). This original strategy of screening limits the number of tags, primers and probes to detect each strain in a pool and thus reduces considerably the costs of screening.

### *In vitro* detection of barcoded *C. albicans* strains

A pool of infection contained ten strains: eight strains of the TF mutant collection, a wild type strain as a positive control and an avirulent strain (a *cmp1*Δ mutant) as a negative control. Each strain of the pool was tagged with a barcode consisting of a unique 40 oligonucleotides sequence (Table 2) selected among STM (Sequence Tag Mutagenesis) tags previously used in *C. neoformans* [38], [39]. Each barcode was cloned in a *C. albicans* optimized plasmid (CIp30) [37] and transformed in TF mutant strains as indicated in Table 1. Ten sets of Taqman probes and primers were designed as presented in Figure 2 for STM6 in order to specifically detect each barcode.

**Figure 3:**
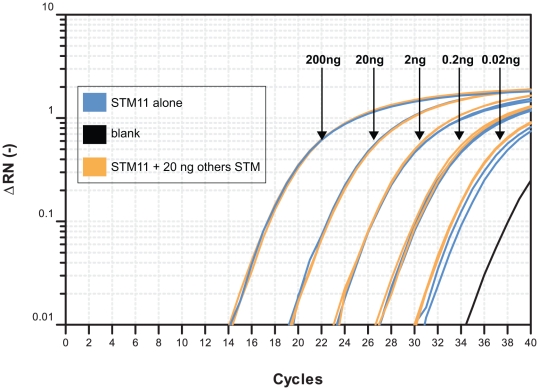
Limit of detection of a given barcoded strain in a pool. Signal curves of qPCR over cycle numbers: samples contain 10 ng of the DNA of nine barcoded strains equally mixed and 10-fold serial dilutions of the DNA (from 200 to 0.02 ng) of the strain with the barcode STM11 (**Orange**). Samples contain only 10-fold serial dilution of the DNA (from 200 to 0.02 ng) of the strain with the barcode STM11 (**Blue**). No template control corresponds to the limit of DNA detection of the assay (**Black**).

Our first attempt was to specifically detect each barcode *in vitro*. For this purpose, the ten STM-CIp30 plasmids were introduced into the wild type strain BWP17. We thus obtained ten strains differing only by their barcodes. Using DNA from *in vitro* culture of each strain and from co-culture of all strains, we were able to detect each strain specifically (data not shown). Our second attempt was to determine the limit of detection of a strain within a pool. The DNAs of the ten BWP17-tagged strains, except the one carrying the STM11-tag, were mixed in an equal amount. The DNA of the STM11-tagged strain was diluted to a concentration of 10 ng/µl and 10-fold serially diluted to reach a concentration of 0.01 ng/µl. Each dilution was mixed with 10 ng of pool DNA. STM11 was detected specifically in the different samples (Figure 3). BWP17-STM11 DNA can be detected even if 500-fold diluted in the pool as compared to other DNAs (Figure 3).

The set of ten barcoded plasmids, primers and probes designed for their detection represents therefore a powerful tool optimized for the *in vitro* qPCR detection of a given *C. albicans* tagged strain within a mixed population.

### Detection of *C. albicans* barcoded strains *in vivo*

The subsequent test was to assess the detection of each barcode *in vivo*. We first determined the threshold above or under which a strain may be considered as colonizing mice organs differently than the wild type. For this purpose a “BWP17 *in vivo* pilot assay” was performed with a pool containing ten BWP17-tagged strains as described above. This *in vivo* pilot assay was performed three times with groups of three mice. At day three post-infection, which is the minimal time for *C. albicans* required to complete a systemic infection [43], mice kidneys were recovered. *C. albicans* DNA extraction from mice kidneys was optimized [44] to reduce contamination by mammalian DNA. In parallel, *C. albicans* DNA was also extracted from a 24 h *in vitro* co-culture in 50 ml YEPD of the same inoculum (250 µl). The relative abundances of each DNA were quantified by qPCR. The detection of each barcode from mice kidneys was successful. The score of infection of each BWP17-tagged strain was calculated relative to that of the BWP17-STM6 strain as described in Material and Methods. When the results of the three independent experiments were pooled, the medium score has a value of −0.05 (± 0.72) (Supplementary File S1). This value, to which ± two standard deviations were added (1.39 to −1.49), corresponds to the threshold above or under which a strain has a different colonization potential as compared to the wild type.

Each pool contains a *cmp1*Δ strain as a negative control of infection. *CMP1* encodes for the catalytic subunit of calcineurin. Previous studies extensively described that strains lacking a functional calcineurin are avirulent and unable to colonize mice organs in the model of disseminated infection [45], [46], [47]. To validate our screening system, we verified whether or not such a strain could be detected from infected mice tissues. For this purpose, we performed a so called “*CMP1*“ pilot experiment, in which a pool containing a BWP17-STM6 tagged strain and nine *cmp1Δ* strains carrying the other barcodes was injected in groups of three mice. The *cmp1Δ* mutants (all tags combined) showed a score of −4.02 (± 2.12) (data not shown), thus confirming that the system was able to detect a strain with a colonization score lower than the wild type. These *in vivo* pilot assays demonstrated that our strategy of screening was functional *in vivo* and also defined the limits of detection and interpretation of the results.

### Analysis of ten pools of Zn2-Cys6 TF mutant strains

The screening of the 77 *C. albicans* Zn2-Cys6 mutant strains in a mouse model of systemic infection was performed by designing ten pools of infection as detailed in Table 1. These 77 mutants correspond to only 74 mutated genes since three genes were mutated twice independently (*i.e.* orf19.5133, orf19.3012 and orf19. 3876) (Supplementary File S1). All mutant strains were transformed with plasmids derived from STMx-CIp30. These plasmids carrying the STM-tags allow also to re-introduce *URA3*, *HIS1* or *ARG4* markers in a neutral locus for virulence [37].

Pool 1 was containing nine strains with a CAF4-2 background (Table 1 and Supplementary File S1). The seven Zn2-Cys6 TFs and the *cmp1*Δ mutants of the pool were constructed using URA blaster cassettes in the CAF4-2 background [48], [49], [50]. Therefore, CAF4-2 containing STM6-CIp30 was the positive control. The negative control was DSY2101 (*cmp1*Δ) containing STM240-CIp30 (Table 1). The nine other pools contained isolates with a BWP17 background. Each pool except pools 2 and 6 contained eight Zn2-Cys6 TF mutants (only seven for pools 2 and 6), BWP17 tagged with STM6 and DSY4343 (*cmp1*Δ) tagged with STM240. Mutants of these pools were obtained using different strategies as detailed in Supplementary File S1.

**Figure 4:**
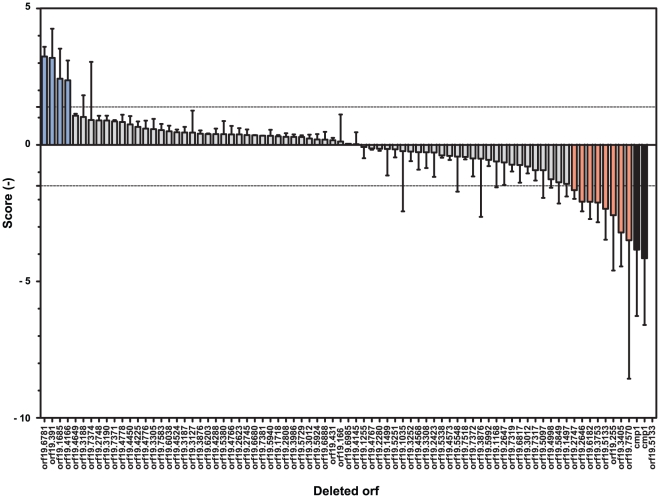
Strain scores of mice kidney colonization. S scores are plotted for each tested mutant. The scores were defined as the values obtained by the equation: S score  =  log2(dQx/dQ_STM6_), where Qx is the relative quantification of the different *C. albicans* DNA as compared to a standard curve included in each qPCR reaction for a given barcode. A first normalization was performed to limit the “pool effect” on the strain growth and the PCRs efficacy effect by the equation dQx =  Qx_INVIVO/_Qx_INVITRO_ with X corresponding to a given barcode. A second normalization was performed based on the wild type strain in order to compare each pool with each other. Since the barcode STM6 was carried by the wild type strain in each pool as positive control, the following equation was applied: dQx/ΔQ_STM6_. The *cmp1*Δ strains carried always the barcode STM240. Orf19.5133 was mutated in two independent strains. Strain BCY158 yielded no result since qPCR signals were under the detection threshold.

Our first analysis was performed on *cmp1*Δ strains. When merging the scores of BCY82 (*cmp1*Δ in the BWP17 background) from pools 2 to 10 and from the “*CMP1* pool” as detailed above, we obtained an average score of −3.83 (± 2.42). It is important to note that in some experiments, no qPCR signals could be obtained for BCY82, probably because the DNA of this strain was under the limit of detection. This means that the DNA of this strain was at least 500-fold more diluted as compared to other DNAs of the pool that included mice DNA from kidneys. BCY13 (*cmp1*Δ mutant in a CAF4-2 background) showed a score of -4.15 (± 2.44) (Figure 4 and Supplementary File S1). Both scores obtained for the *cmp1*Δ strains (BCY82 and BCY13) were under the threshold value of −1.49 as mentioned above, thus confirming that these strains are less colonizing mice tissues than the wild type independently of the genetic background. After setting the detection limits of our *in vivo* screening system, we determined the colonization scores of all strains of the Zn2-Cys6 TF mutant collection in comparison to their isogenic wild type strains. The scores of all strains are presented in Figure 4 and detailed in Supplementary File S1. The majority of the strains have a score within the interval delimited by the “BWP17 pool” assay, indicating that their colonization scores were not significantly different from the wild type strains. In contrast, four strains (as referred to hyper-colonizers), *i.e.* BCY27, BCY150, BCY160 and BCY162 showed scores of 2.36, 3.23, 2.42 and 3.18, respectively, and were above the “BWP17 pool” limit. This suggests a higher colonization capacity than the wild type. Next, eight strains (as referred to hypo-colonizers), *i.e.* BCY36, BCY21, BCY164, BCY88, BCY112, BCY122, BCY148 and BCY152 showed scores below the “BWP17 pool” limit (−3.2, −2.57, −2.34, −1.65, −3.49, −2.08, −2.11, and −2.08, respectively). These data suggest that these strains are poor colonizers as compared to the wild type.

The four hyper-colonizers strains correspond to mutations of orf19.4166, orf19.6781, orf19.1685 and orf19.391. The orf19.4166 (*ZCF21*), orf19.6781 (*ZFU2*) and orf19.1685 (*ZCF7*) encode TFs with yet unknown functions. Interestingly, orf19.391 (also called *UPC2)* encodes a TF regulating genes of the *ERG* family involved in sterol biosynthesis and therefore plasma membrane integrity. Upc2 was also reported to be involved in antifungal resistance due to acquisition of gain-of-function mutations [51], [52], [53], [54].

The eight hypo-colonizer strains correspond to mutations of orf19.3405 (*ZCF18*), orf19.255 (*ZCF1*), orf19.5133 (*ZCF29*), orf19.2747 (*RGT1*), orf19.7570 (*UGA3*), orf19.6182 (*ZCF34*), orf19.3753 (*SEF1*) and orf19.2646 (*ZCF13*). Interestingly, orf19.5133 encodes a TF with yet unknown function and is deleted and interrupted in strains BCY164 (pool 3) and BCY158 (pool 10), respectively. BCY164 (orf19.5133) contained the tag STM209 and showed a score of −2.34 (± 1.13). BCY158, tagged with STM224, did not appear as hypo-colonizer since no signal above the background was obtained by qPCR for this strain.

Five other genes including orf19.3405, orf19.255, orf19.6182, *UGA3* and orf19.2646 encode Zn2-Cys6 TF with yet unknown function. These TFs have been analysed in large scale screening of mutants or found as target genes in transcriptional microarray analyses [22], [24], [42], [55]. *RGT1* encodes a transcriptional repressor involved in the regulation of glucose transporter genes [56]. *SEF1* encodes a TF involved in iron assimilation and survival in stationary phase at 30°C [57], [58].

### Single strain infections

**Figure 5:**
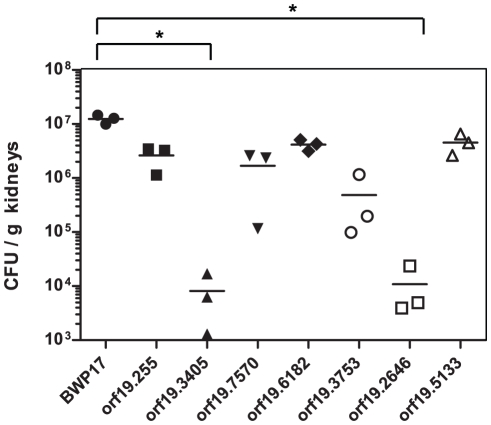
Single strain infections. Seven strains with the lowest scores of colonization in the pool experiments were tested for kidney colonization by single strain mice infection. Groups of three mice were infected with a unique strain. Three days after infection, kidneys were harvested and CFU were measured. Symbol (*) indicates a p-value ≤ 0.05 by the Kruskal-Wallis test.

In order to validate our previous results and in order to eliminate false positives due to pool effect in the infections, we performed single strain infection with seven hypo-colonizers and BCY31 (BWP17 tagged with STM6). As in the case of pools experiments, groups of 3 mice were infected intravenously with 5×10^5^ cells of *C. albicans* mutant strains. Kidney colonization was next analysed by counting *C. albicans* CFUs in kidneys at three days post-infection as performed in the pool experiments. Results are presented in Figure 5. We observed that fungal burdens of all mutant strains were lower than the wild type. Nevertheless, only mutants for orf19.3405 and orf19.2646 showed a significant statistical difference in tissue colonization as compared to BCY31 (8.11×10^3^ and 1.09×10^4^ CFU/g kidney for mutants and 1.24×10^7^ for BCY31). Therefore, these two mutants constitute interesting candidates for further analyses.

### *In vitro* phenotypes of Zn2-Cys6 TF mutants

In an attempt to correlate *in vivo* results with putative functions of identified TFs, the whole collection was screened for six phenotypes potentially relevant for *in vivo* growth by comparing each mutant with its parent wild type strain. First, we determined resistance to heat and oxidative stress by incubation at 42°C and in H_2_O_2_-supplemented medium, respectively, and growth capacities at alkaline or acidic pH of each mutant on rich media. Considering the environmental conditions that *C. albicans* faces within the host, an enhanced or reduced resistance to heat-, oxidative- or pH-stresses may give indications regarding the involvement of a given Zn2-Cys6 TF in virulence and/or colonization abilities. Since switching from the yeast to hyphal form is known to be a determinant of virulence [59], the ability of each mutant to produce true hyphae and/or pseudohyphae was also assessed. For that purpose, colony wrinkling was visually inspected after 72 h of incubation on rich media supplemented with 10% serum, which is known to induce filamentation in *C. albicans* [59]. Moreover, agar invasion was quantified by washing YEPD agar plates after 48 h of incubation at 35°C and by visually evaluating a decrease or an increase of invasive growth as compared to the wild type strain. Raw pictures of this screening are provided as supplementary data (see Supplementary File S2, S3, S4, S5, S6, S7). Lastly, pictures of cells grown for 4 h in YEPD liquid media supplemented with 10% FCS were analyzed for the presence of true hyphae.

**Figure 6:**
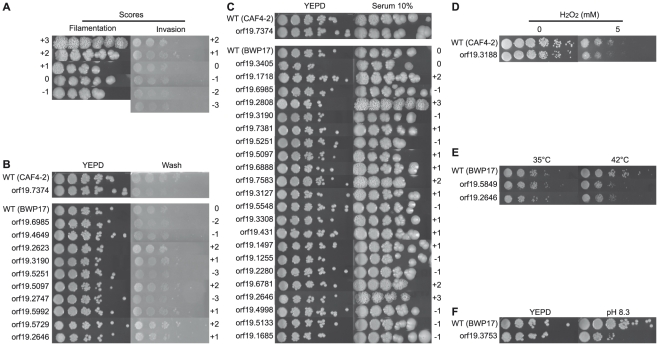
Filamentation, invasive growth and susceptibility to heat, oxidative and pH stresses of the Zn2-Cys6 TF mutants. Pictures of serial dilutions assay plates for the Zn2-Cys6 TF mutants displaying an *in vitro* phenotype (see Supplementary File S1 for phenotype details) were taken after 24, 48 or 72 h of incubation. Mutants are referenced on the left side of each panel according to the disrupted ORF number (see Supplementary File S1). Conditions of incubation are indicated on the top of the figure. (**A**) Reference panel attributing a score relative to wild type phenotype (score 0), is given for better visualization of differences between BWP17-based mutants and wild type regarding (**B**) invasion and (**C**) filamentation phenotypes. Since only one filamentation and one invasion altered phenotypes were observed for CAF4-2-based mutants (i.e mutant for orf19.7374), such a reference panel was not included. (**D**) Growth of orf19.3188 (*TAC1*) mutant on H_2_O_2_-containing medium. (**E**) Mutants with reduced growth at 42°C or (**F**) alkaline pH. The phenotypes of each mutant were compared with its respective parental strain, which was spotted on each plate for reference.

Overall, 39 out of 74 mutants displayed an altered phenotype regarding the six criteria tested here (Tables 3, Figure 6 and Supplementary File S1, sheet “phenotype”). Only a few mutants displayed altered susceptibilities to heat, pH and H_2_O_2_. Indeed, none of the mutants showed impaired growth at acidic pH and only one of them, *SEF1*, exhibited decreased growth at alkaline pH. Likewise, only one mutant, orf19.3188 (*TAC1*) was hypersusceptible to H_2_O_2_. Two mutants had a decreased growth capacity at 42°C, namely orf19.5849 (*CWT1*), and orf19.2646 (*ZCF13*). The largest phenotypic alterations were observed in morphology-discriminative conditions since among the 39 mutants displaying a phenotype in our screen, 28 were selected for their abnormal morphology on solid YEPD 10% FCS media and/or their altered ability to invade agar (Figure 6 and Supplementary file S1, sheet “phenotype”). Out of 22 mutants with abnormal morphology, 13 produced less wrinkled colonies as compared to their respective wild-type parents and 9 an increased colony wrinkling. Regarding agar invasion ability, 6 and 5 mutants showed increased and decreased invasion in the solid substrate, respectively, as compared to the wild-type (Figure 6 and Table 3).

**Table 3:** ORFs deleted in the Zn2Cys6 TF mutants with altered phenotypes on solid media as compared to wild type strain.

Altered phenotype								
Increased susceptibility to			Agar invasion		Colony wrinkling		Hyphae formation	
Heat	Oxidative stress	Alkaline pH	Increased	Decreased	Increased	Decreased	Increased	Decreased
orf19.2646	orf19.3188	orf19.3753	orf19.2623	orf19.2447	orf19.431	orf19.1255	orf19.1168	orf19.166
orf19.5849			orf19.2646	orf19.4649	orf19.1497	orf19.1685	orf19.3305	orf19.1685
			orf19.3190	orf19.5251	orf19.1718	orf19.2280	orf19.3405	orf19.2647
			orf19.5097	orf19.6985	orf19.2646	orf19.3190	orf19.4649	orf19.2747
			orf19.5729	orf19.7374	orf19.2808	orf19.4998	orf19.4766	orf19.3188
			orf19.5992		orf19.3127	orf19.5133	orf19.5097	orf19.3753
					orf19.3308	orf19.5251	orf19.5729	orf19.3986
					orf19.5097	orf19.5548	orf19.6038	orf19.4998
					orf19.6781	orf19.6985	orf19.6985	orf19.5133
					orf19.6888	orf19.7374	orf19.7381	orf19.5849
					orf19.7381			orf19.7374
					orf19.7583			

Liquid YEPD 10% FCS media cultures allowed the selection of 21 mutants with a modified cell morphology as compared to their parental wild type strains. Mutants with more than 80% of cells forming hyphae were scored as positive. In contrast, mutants showing less than 10% of hyphae were scored as negative (see Table 3 and Supplementary file S1, sheet “Phenotype”). Following these criteria, 11 mutants displayed almost no hyphae formation. In contrast, 10 displayed an increase in true hyphae formation.

*In vivo* single infection experiments allowed the selection of two interesting TF (orf19.2646 and orf19.3405) mutant strains for their hypo-colonizer phenotype in kidneys. Mutant for orf19.2646 produced highly wrinkled colonies on YEPD agar medium supplemented with 10% FCS and also better invaded the agar as compared to the wild-type. Mutant for orf19.3405 showed a growth deficiency in all conditions tested (Figure 6) but displayed an increased production of true hyphae in liquid YEPD supplemented with 10% FCS (Table 3 and Supplementary file S1).

### Reversion of the orf19.2646 deletion phenotypes

**Figure 7:**
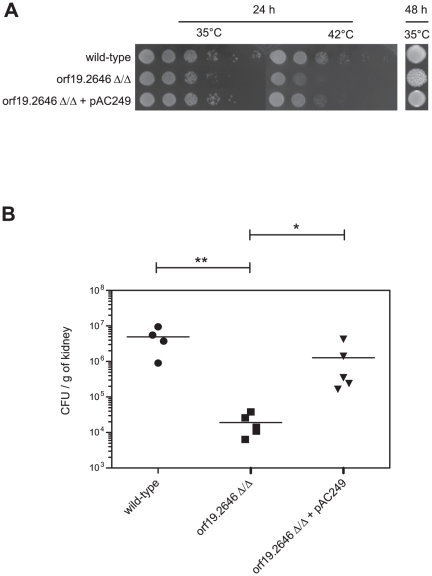
Phenotypes of orf19.2646 mutant and revertant strains. (**A**) *In vitro* growth in YEPD agar medium at either 35 or 42°C for 24 h. Enhanced resolution picture was taken after 48 h of incubation at 35°C to better visualize the wrinkled colony phenotype. (**B**) *In vivo* colonization assay. Groups of four to five mice were infected with either wild-type, orf19.2646 mutant or revertant strains. Three days after infection, kidneys were harvested and CFU were measured. Symbol (*) and (**) indicate a p-value ≤ 0.05 and 0.01, respectively, as determined by the Kruskal-Wallis test.

We further investigated orf19.2646 since it appeared as a promising candidate to discover yet unknown virulence factors. To confirm that both *in vitro* and *in vivo* phenotypes observed were effectively due to the interruption of orf19.2646, we constructed a revertant strain by re-introduction of a wild-type gene at the genomic locus in the BCY152 background (see Material and Methods). As shown in Figure 7A, the revertant strain exhibited as the parent wild-type no growth deficiency at 42°C and no longer displayed a wrinkled colony morphology in contrast to the mutant strain. Likewise, animal experiments confirmed that the re-introduction of orf19.2646 restored a colonization capacity comparable to the wild-type strain which was also statistically different from the mutant (Figure 7B).

## Discussion

In this work, we screened at large scale the role of *C. albicans* Zn2-Cys6 TFs in organs colonization using a mouse model of systemic infection. For this purpose, we developed a screening strategy infecting mice with pools of ten strains tagged with a set of ten barcodes consisting of 40-nucleotides sequences. Among the 77 Zn2-Cys6 TF mutants tested corresponding to 74 mutated ORF, four resulted in a hyper-colonizer phenotype while eight showed hypo-colonizer phenotype (see Supplementary file S1). Recently, similar large-scale *in vivo* mutant screening were performed by Noble *et al.* [24] and Chamilos *et al.* [55]. This last work was performed in the Toll mutant fly model, and focused on 33 TF mutants with no restriction on the TF class [55]. The mutants used came from the same TF mutant collection as used here. Twelve mutants were common between this study and ours (see Supplementary file S1, “all-scores” sheet). Among these twelve mutants, none were found hypo-colonizer in the fly model and only one (*UGA3*) was found hypo-colonizer in our pool experiment. The use of two distinct infection models might explain the discrepancies between both studies.

The work of Noble *et al.* [24] was more similar to our analysis since mutant strains of this study were barcoded and were infecting mice as pools. Nevertheless, our analyses were different since Noble *et al.* [24] did not target a specific class of genes and developed their own strategy of screening associated to their particular mutant collection [24]. Consequently, out of the 77 Zn2-Cys6 TF mutants tested here, only 26 were common with the study of Noble *et al.* [24] (see Supplementary file S1, “all-scores” sheet). Three of the eight hypo-colonizers mutants, namely mutants for orf19.255 (*ZCF1*), orf19.6182 (*ZCF34*) and *SEF1*, were also tested in their study. Only the mutant for *SEF1* showed a hypo-colonizer phenotype in both studies (Table 4). Transcriptional analyses on iron metabolism in *C. albicans* showed that *SEF1* might be involved in iron uptake regulation [58]. Interestingly, Chen *et al.* [60] demonstrated recently that *SEF1*, as observed here (Table 4), is critical for *in vivo* colonization. The *SEF1* deletion was accompanied by a decreased virulence in the mice bloodstream infection model [60]. Fortunately, none of the mutants found as hypo-colonizers by Noble *et al.* [24] were missed using our strategy. Besides, none of the four mutants that were found hyper-colonizers were identified by Noble *et al.* [24]. To our knowledge, the remaining four mutants found here as hypo-colonizer including mutants for orf19.5133 (*ZCF29*), orf19.3405 (*ZCF18*), orf19.2646 (*ZCF13*) and *RGT1* have not yet been identified in studies addressing virulence or organ colonization in animal studies.

**Table 4:** *In vitro* phenotypes of *C. albicans* Zn2-Cys6 TF mutants with hypo- or hyper-colonization phenotype in a mice model of disseminated candidiasis.

ORF number	Gene name	Phenotype in our study	Other phenotypes
Hyper- colonizers			
19.6781	*ZFU2*	increased colony wrinkling on serum	decreased invasive growth [69]
			weak enhancement of invasion of YEPD medium [22]
19.1685	*ZCF7*	decreased colony wrinkling on serum	impaired growth on Lee's medium [22]
		no hyphae formation in liquid YEPD serum	reduced colony wrinkling on Spider medium [22]
19.391	*UPC2*	no phenotype	accumulation of abnormal sterol intermediates [53]
			hypersusceptible to azole antifungals [53]
			decreased anaerobic growth rate [70]
			haploinsufficiency on SLAD medium [69]
19.4166	*ZCF21*	no phenotype	weak resistance to Caffeine [22]
			weak resistance to Copper at 37°C [22]
			marginal sensitivity to Calcofluor White [22]
Hypo- colonizers			
19.3405	*ZCF18*	growth-deficient on YEPD media	hypersusceptible to virgineone [71]
		increased hyphae formation in liquid YEPD serum	
19.255	*ZCF1*	no phenotype	no phenotype
19.5133	*ZCF29*	decreased colony wrinkling on YEPDA serum	strong sensitivity to Caffeine and Menadione [22]
		no hyphae formation in liquid YEPD serum	strong resistance to Fenpropimorph [22]
19.2747	*RGT1*	decreased invasive growth	decreased resistance to 2-deoxy-D-glucose [67]
		no hyphae formation in liquid YEPD serum	increased filamentous growth [56], [67]
			strong reduction of colony wrinkling at 37°C and 42°C [22]
19.7570	*UGA3*	no phenotype	no phenotype
19.6182	*ZCF34*	no phenotype	increased susceptibility to clotrimazole [69]
			increased mortality rate in stationary growth phase [57]
			abnormal morphology on solid Spider medium [24]
19.3753	*SEF1*	hypersusceptibility to alkaline pH	increased mortality rate in stationary growth phase [57]
		no hyphae formation in liquid YEPD serum	strong sensitivity to elevated pH, weak sensitivity to SDS [22]
			haploinsufficiency on SLAD medium [69]
			decreased colonization [24], [60]
19.2646	*ZCF13*	increased colony wrinkling and invasion	no phenotype
		hypersusceptibility to heat	

To further characterize the putative function of the Zn2-Cys6 TFs deleted in our study, we screened the whole collection for altered hyphae formation and colony morphology on serum, invasive growth and susceptibility to pH, heat and oxidative stresses. These six phenotypic criteria are known to play a major role in *C. albicans* pathogenesis (for review see [59]). *SEF1* mutant had an impaired growth at alkaline pH, which has already been described by Lan *et al.* [58]. Only one mutant displayed altered susceptibility to H_2_O_2_. Interestingly, this mutant was *TAC1* (orf19.3188), the main regulator of the expression of genes encoding transporters belonging to the ATP-binding cassette family, which are known to mediate azole resistance [48]. Liu *et al.* found that *TAC1* is regulating some genes involved in oxidative stress response such as *GPX1* and *SOD5* [61]. Moreover several studies suggested that azole drugs are able to generate an oxidative stress through mitochondrion metabolism disturbance [62], [63], [64]. Our observation further reinforces this hypothesis.

Lastly, only two mutants were hypersusceptible to heat stress, namely mutants for orf19.5849 (*CWT1*) and orf19.2646 (*ZCF13*). To our knowledge, and without any further investigations, no obvious correlation between impaired growth at 42°C and the putative functions of these TFs could be observed.

Contrary to pH, oxidative and heat stresses, most of the phenotypes altered in our TF mutants were hyphae production in liquid medium supplemented with serum, morphological aspect of colonies, and invasive growth (Table 3, Figure 6 and Supplementary file S1). In *C. albicans*, several parameters are known to induce hyphal formation (serum and temperature as example) however the mechanisms by which this yeast can sense contact with a surface and can promote invasion of soft substrates like agar medium or host tissue are still not fully understood [65]. However, invasive growth and colony wrinkling are two partially independent mechanisms (or at least mechanisms mediated by different effectors) helping *C. albicans* to cope with the requirements of its environment. Our results further demonstrate that agar invasion, colony wrinkling, and hyphae formation could not be correlated since a high discrepancy was observed while performing *in vitro* phenotypic screening of our TF mutants collection.

**Figure 8:**
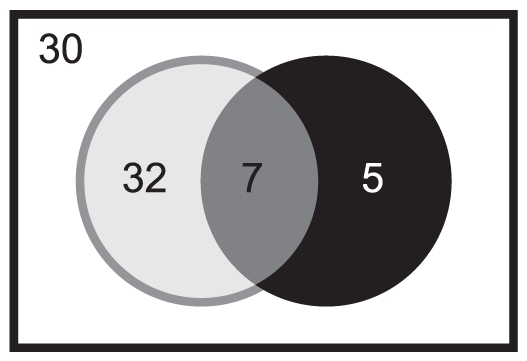
Venn diagram of *C. albicans* Zn2-Cys6 TF mutants with an *in vitro* and/or an *in vivo* phenotype. The 49 mutants (**grey circle**) with altered morphology and/or invasive growth, or altered susceptibility to pH, oxidative and/or heat stresses as determined by *in vitro* serial dilutions assay and liquid culture were compared to the 12 mutants with increased or decreased mice kidney colonization capacity (**black circle**). The remaining 22 mutants (**white rectangle**) did not have either *in vivo* or *in vitro* phenotypes.

More than the hyphal form itself, the ability of *C. albicans* to switch from yeast to hyphae or pseudohyphae is of crucial importance for its virulence [66]. Accordingly, no correlation between colony wrinkling and/or hyphae formation in liquid medium and reduced organs colonization could be made from our results (Figure 8). Indeed, only four of our hypo-colonizer mutants (orf19.5133, orf19.2747, orf19.3753, and orf19.1685) have a reduced hyphae production. Likewise, only one hypo-colonizer mutant lost its capacity to invade agar (orf19.2747), and only two (orf19.5133 and orf19.1685) displayed smooth colonies. Even mutant for orf19.2646 and orf19.3405, although compromized in organ colonization, produced highly wrinkled colonies on serum-supplemented media and a larger amount of hyphae in the presence of serum, respectively, as compared to the wild type (Table 3). Likewise, no correlation between increased filamentation on serum and colonization ability could be evidenced since all but one (orf19.6781) of the hyper-colonizer mutants produce highly wrinkled colonies on serum-supplemented media (Figure 6 and Table 3). This enhanced ability to produce hyphae may explain partially its higher ability to colonize mice tissues.

One unexpected observation was the poor correlation between decreased invasive growth and hypo-colonizer phenotypes as only two hypo-colonizer TF mutants (for *RGT1* and *CTA4*) displayed a decreased agar invasion (Figure 6 and Table 4). This result may appear surprising since it is well established that invasion ability is a crucial determinant for the capacity of *C. albicans* to colonize host tissues [66]. *RGT1* was previously extensively analysed *in vitro* for its role in sugar transport and metabolism [56], [67], thus the decreased colonization ability of *RGT1* mutant might not be only linked to its decreased invasion capacity but probably to a combination with sugar metabolism. The *CTA4* mutant, which also produced smooth colonies on serum-supplemented media and no hyphae in liquid media, has been described with a decreased virulence by Chiranand *et al.* [68]. Even if virulence and colonization are two distinct mechanisms of pathogenesis, it would be interesting to address whether this mutant also exhibits decreased virulence in the mice model of disseminated infection.

In conclusion, the reduced or enhanced ability of most of the identified mutants to colonize mice kidneys could be at least partially explained by their *in vitro* phenotype (Table 4). However, a systematic relationship between criteria investigated here and colonization ability could be ruled out from our results. Indeed, among the 39 TF mutants positively selected from our *in vitro* test, only 7 had a modified colonization capacity in mice as compared to their wild type strains (Figure 8). Moreover, five of these twelve TF mutants did not have any *in vitro* phenotype, suggesting that their increased or decreased colonization ability could be attributed to alternative virulence traits.

Finally, to validate results obtained with hypo-colonizer mutants, single strain infections were performed with the seven hypo-colonizer mutants and the wild type strain. Even if all mutants showed decreased colonization as compared to wild type, only two mutants for orf19.2646 (*ZCF13*) and orf19.3405 (*ZCF18*) showed significant reduction of colonization by CFU counting in kidneys as compared to the wild type. Surprisingly, these two strains did not show the lowest colonization scores in pool experiments. This result can be explained by a “pool effect”, which might influence the colonization of each strain of the pool. Competition between strains can modify the behavior of a strain in mice organs as compared to single strain infection. Our *in vitro* analysis showed that the mutant for orf19.3405 (*ZCF18*) is slightly growth-deficient, and thus might contribute to its hypo-colonizer phenotype. Regarding mutant for orf19.2646, we observed an increased ability to filament and to invade the agar and a growth deficiency at 42°C. The phenotypes observed *in vitro* and *in vivo* were reverted by the re-introduction of a wild-type allele in the mutant strain confirming that phenotypes observed were effectively due to the orf19.2646 (*ZCF13)* mutation. Orf19.2646 (*ZCF13*) therefore represents a very interesting candidate for further analyses. The further characterization of the function of this TF in virulence and identification of its target genes may constitute promising basis for the better understanding of host-*Candida* interactions. In addition, analysis of the remaining mutants of our collection may reveal other interesting candidates for involvement in colonization and virulence.

Overall, our study demonstrates that our original strategy is a powerful tool to detect both *in vivo* and *in vitro* competitive fitness. This last *in vitro* approach is currently used in our laboratory. Moreover, this detection system is easy to implement, could be adapted to any pre-existing mutants and its efficacy could be enhanced by the use of additional STM tags.

## Supporting Information

Supplementary S1 This file contains five sheets. The “Zn2Cys6 TF” sheet describes the mutant collection and provides for each mutant ORF number, gene name (when available) and description according to the *Candida* Genome Database assembly #21. It also provides information for inactivation strategy and numbering of barcoded strains (BCY). The “Phenotype” sheet describes the phenotype that was observed *in vitro* and a summary of the already described phenotypes for each mutant. The “All scores” sheet gives scores obtained for each replicate of *in vivo* experiments as well as the mean score for each mutant (see also Figure 4). It also gives information regarding previous large scale *in vivo* studies. The “BWP17 pilot assay” gives scores obtained for each replicate of the first *in vivo* pilot assay performed with the 10 BWP17 barcoded strains. Results are presented by STM. The “Supplementary references” sheet lists the references used in the “Phenotype” sheet.
(XLSX)

Supplementary S2 Raw pictures of first *in vitro* phenotypic screen plates after 24 h of incubation. Each TF mutant is referenced according to its barcoded strain number (see Supplementary_S1.xlsx), and has to be compared with its respective parental strain (barcoded strain number 11 for CAF4-2 based TF mutants and barcoded strain number 31 for BWP17 based TF mutants) which was spotted on each plate for reference. Note that susceptibility to H_2_O_2_ was only determined at 5 mM.
(EPS)

Supplementary S3 Raw pictures of first *in vitro* phenotypic screen plates after 48 h of incubation. Each TF mutant is referenced according to its barcoded strain number (see Supplementary_S1.xlsx), and has to be compared with its respective parental strain (barcoded strain number 11 for CAF4-2 based TF mutants and barcoded strain number 31 for BWP17 based TF mutants) which was spotted on each plate for reference.
(EPS)

Supplementary S4 Raw pictures of second *in vitro* phenotypic screen plates after 24 h of incubation. Each TF mutant is referenced according to its barcoded strain number (see Supplementary_S1.xlsx), and has to be compared with its respective parental strain (barcoded strain number 11 for CAF4-2 based TF mutants and barcoded strain number 31 for BWP17 based TF mutants) which was spotted on each plate for reference. H_2_O_2_ concentration was adjusted and susceptibility of TF mutants was determined at 1 and 5 mM.
(EPS)

Supplementary S5 Raw pictures of second *in vitro* phenotypic screen plates after 48 h of incubation. Each TF mutant is referenced according to its barcoded strain number (see Supplementary_S1.xlsx), and has to be compared with its respective parental strain (barcoded strain number 11 for CAF4-2 based TF mutants and barcoded strain number 31 for BWP17 based TF mutants) which was spotted on each plate for reference.
(EPS)

Supplementary S6 Raw pictures of additional *in vitro* phenotypic screen performed for morphology determination. Each TF mutant is referenced according to its barcoded strain number (see Supplementary_S1.xlsx), and has to be compared with its respective parental strain (barcoded strain number 11 for CAF4-2 based TF mutants and barcoded strain number 31 for BWP17 based TF mutants) which was spotted on each plate for reference. This was performed on YEPD agar plates supplemented with 10% of serum after 24 and 72 h of incubation at 35°C.
(EPS)

Supplementary S7 Raw pictures of *in vitro* phenotypic screen for TF mutants carrying the barcoded strain numbers BCY48, BCY122, BCY124, BCY126, BCY166 and BCY164. These strains were obtained in a second time during the course of this study and therefore assayed independently from the main screen. Each TF mutant is referenced according to its barcoded strain number (see Supplementary_S1.xlsx), and has to be compared with the parental strain (barcoded strain number 31) which was spotted on each plate for reference.
(EPS)

## References

[1] Romani L, Bistoni F, Puccetti P (2003). Adaptation of Candida albicans to the host environment: the role of morphogenesis in virulence and survival in mammalian hosts.. Curr Opin Microbiol.

[2] Hube B (2004). From commensal to pathogen: stage- and tissue-specific gene expression of Candida albicans.. Curr Opin Microbiol.

[3] Sanglard D, Odds FC (2002). Resistance of Candida species to antifungal agents: molecular mechanisms and clinical consequences.. Lancet Infect Dis.

[4] Akins RA (2005). An update on antifungal targets and mechanisms of resistance in Candida albicans.. Med Mycol.

[5] Ghannoum MA, Rice LB (1999). Antifungal agents: mode of action, mechanisms of resistance, and correlation of these mechanisms with bacterial resistance.. Clin Microbiol Rev.

[6] White T, Marr K, Bowden R (1998). Clinical, cellular, and molecular factors that contribute to antifungal drug resistance.. Clin Microbiol Rev.

[7] Sanglard D, Coste A, Ferrari S (2009). Antifungal drug resistance mechanisms in fungal pathogens from the perspective of transcriptional gene regulation.. FEMS Yeast Res.

[8] Chandra J, Kuhn D, Mukherjee P, Hoyer L, McCormick T (2001). Biofilm formation by the fungal pathogen Candida albicans: development, architecture, and drug resistance.. J Bact.

[9] Ramage G, Bachmann S, Patterson T, Wickes B, Lopez-Ribot J (2002). Investigation of multidrug efflux pumps in relation to fluconazole resistance in Candida albicans biofilms.. J Antimicrob Chemother.

[10] Sellam A, Tebbji F, Nantel A (2009). Role of Ndt80p in sterol metabolism regulation and azole resistance in Candida albicans.. Eukaryot Cell.

[11] Netea MG, Maródi L (2010). Innate immune mechanisms for recognition and uptake of Candida species.. Trends in Immunology.

[12] Bourgeois C, Majer O, Frohner I, Tierney L, Kuchler K (2010). Fungal attacks on mammalian hosts: pathogen elimination requires sensing and tasting.. Curr Opin Microbiol.

[13] van de Veerdonk F, Kullberg B, Netea M (2010). Pathogenesis of invasive candidiasis.. Curr Opin Crit Care.

[14] van de Veerdonk F, Netea M, Joosten L, Van der Meer J, Kullberg B (2010). Novel strategies for the prevention and treatment of Candida infections: the potential of immunotherapy.. FEMS Microbiol Rev.

[15] Davis DA (2009). How human pathogenic fungi sense and adapt to pH: the link to virulence.. Curr Opin Microbiol.

[16] Almeida R, Wilson D, Hube B (2009). Candida albicans iron acquisition within the host.. FEMS Yeast Res.

[17] Klis F, Sosinska G, de Groot P, Brul S (2009). Covalently linked cell wall proteins of Candida albicans and their role in fitness and virulence.. FEMS Yeast Res.

[18] Brown AJP, Odds FC, Gow NAR (2007). Infection-related gene expression in Candida albicans.. Curr Opin Microbiol.

[19] Hube B (2006). Infection-associated genes of Candida albicans.. Future Microbiol.

[20] Román E, Arana DM, Nombela C, Alonso-Monge R, Pla J (2007). MAP kinase pathways as regulators of fungal virulence.. Trends Microbiol.

[21] Thewes S, Kretschmar M, Park H, Schaller M, Filler SG (2007). In vivo and ex vivo comparative transcriptional profiling of invasive and non-invasive Candida albicans isolates identifies genes associated with tissue invasion.. Mol Microbiol.

[22] Homann OR, Dea J, Noble SM, Johnson AD (2009). A Phenotypic Profile of the Candida albicans Regulatory Network.. PLoS Genet.

[23] Becker JM, Kauffman SJ, Hauser M, Huang L, Lin M (2010). Pathway analysis of Candida albicans survival and virulence determinants in a murine infection model.. Proc Natl Acad Sci USA.

[24] Noble SM, French S, Kohn LA, Chen V, Johnson AD (2010). Systematic screens of a Candida albicans homozygous deletion library decouple morphogenetic switching and pathogenicity..

[25] Boon J, Lambert T, Sisson A, Davis A, Smith B (2003). Facilitated phosphatidylserine (PS) flip-flop and thrombin activation using a synthetic PS scramblase.. JAmChemSoc.

[26] Davis D, Edwards J, Mitchell A, Ibrahim A (2000). Candida albicans RIM101 pH response pathway is required for host-pathogen interactions.. Infect Immun.

[27] Ikeda F, Wakai Y, Matsumoto S, Maki K, Watabe E (2000). Efficacy of FK463, a new lipopeptide antifungal agent, in mouse models of disseminated candidiasis and aspergillosis.. Antimicrob Agents Chemother.

[28] Moye-Rowley WS (2002). Transcription factors regulating the response to oxidative stress in yeast.. Antioxid Redox Signal.

[29] Felk A, Kretschmar M, Albrecht A, Schaller M, Beinhauer S (2002). Candida albicans hyphal formation and the expression of the Efg1-regulated proteinases Sap4 to Sap6 are required for the invasion of parenchymal organs.. Infect Immun.

[30] Lachke S, Srikantha T, Soll D (2003). The regulation of EFG 1 in white-opaque switching in Candida albicans involves overlapping promoters.. Mol Microbiol.

[31] Staib P, Kretschmar M, Nichterlein T, Hof H, Morschhauser J (2002). Transcriptional regulators Cph1p and Efg1p mediate activation of the Candida albicans virulence gene SAP5 during infection.. Infect Immun.

[32] Kiesewetter DO, Finn RD, Rice KC, Monn JA (1990). Synthesis of 11C-labeled (+-)-5-methyl-10,11-dihydro-5H-dibenzo[a,d]cyclohepten-5,10-imin e [(+-)-[11C]MK801].. Int J Rad Appl Instrum A.

[33] Johnston D, Eberle K, Sturtevant J, Palmer G (2009). Role for endosomal and vacuolar GTPases in Candida albicans pathogenesis.. Infect Immun.

[34] Fonzi W, Irwin M (1993). Isogenic strain construction and gene mapping in Candida albicans.. Genetics.

[35] Wilson R, Davis D, Mitchell A (1999). Rapid hypothesis testing with Candida albicans through gene disruption with short homology regions.. J Bact.

[36] Sanglard D, Ischer F, Monod M, Bille J (1996). Susceptibilities of Candida albicans multidrug transporter mutants to various antifungal agents and other metabolic inhibitors.. Antimicrob Agents Chemother.

[37] Dennison P, Ramsdale M, Manson C, Brown A (2005). Gene disruption in Candida albicans using a synthetic, codon-optimised Cre-loxP system.. Fungal Genet Biol.

[38] Liu O, Chun C, Chow E, Chen C, Madhani H (2008). Systematic genetic analysis of virulence in the human fungal pathogen Cryptococcus neoformans.. Cell.

[39] Moyrand F, Lafontaine I, Fontaine T, Janbon G (2008). UGE1 and UGE2 regulate the UDP-glucose/UDP-galactose equilibrium in Cryptococcus neoformans.. Eukaryot Cell.

[40] Bruno VM, Kalachikov S, Subaran R, Nobile CJ, Kyratsous C (2006). Control of the C. albicans cell wall damage response by transcriptional regulator Cas5.. PLoS Pathog.

[41] Nobile C, Andes D, Nett J, Smith F, Yue F (2006). Critical role of Bcr1-dependent adhesins in C. albicans biofilm formation in vitro and in vivo.. PLoS Pathog.

[42] Nobile C, Mitchell A (2005). Regulation of cell-surface genes and biofilm formation by the C. albicans transcription factor Bcr1p.. Curr Biol.

[43] MacCallum DM, Odds FC (2005). Temporal events in the intravenous challenge model for experimental Candida albicans infections in female mice.. Mycoses.

[44] Moyrand F, Fontaine T, Janbon G (2007). Systematic capsule gene disruption reveals the central role of galactose metabolism on Cryptococcus neoformans virulence.. Molecular Microbiology.

[45] Bader T, Bodendorfer B, Schröppel K, Morschhäuser J (2003). Calcineurin is essential for virulence in Candida albicans.. Infect Immun.

[46] Blankenship J, Wormley F, Boyce M, Schell W, Filler S (2003). Calcineurin is essential for Candida albicans survival in serum and virulence.. Eukaryot Cell.

[47] Sanglard D, Ischer F, Marchetti O, Entenza J, Bille J (2003). Calcineurin A of Candida albicans: involvement in antifungal tolerance, cell morphogenesis and virulence.. Mol Microbiol.

[48] Coste AT, Karababa M, Ischer F, Bille J, Sanglard D (2004). TAC1, transcriptional activator of CDR genes, is a new transcription factor involved in the regulation of Candida albicans ABC transporters CDR1 and CDR2.. Eukaryot Cell.

[49] Coste AT, Ramsdale M, Ischer F, Sanglard D (2008). Divergent functions of three Candida albicans zinc-cluster transcription factors (CTA4, ASG1 and CTF1) complementing pleiotropic drug resistance in Saccharomyces cerevisiae.. Microbiology.

[50] Talibi D, Raymond M (1999). Isolation of a putative Candida albicans transcriptional regulator involved in pleiotropic drug resistance by functional complementation of a pdr1 pdr3 mutation in Saccharomyces cerevisiae.. J Bact.

[51] Hoot S, Oliver B, White T (2008). Candida albicans UPC2 is transcriptionally induced in response to antifungal drugs and anaerobicity through Upc2p-dependent and -independent mechanisms.. Microbiology.

[52] Hoot S, Brown R, Oliver B, White T (2010). The UPC2 promoter in Candida albicans contains two cis-acting elements that bind directly to Upc2p, resulting in transcriptional autoregulation.. Eukaryot Cell.

[53] Silver PM, Oliver BG, White TC (2004). Role of Candida albicans transcription factor Upc2p in drug resistance and sterol metabolism.. Eukaryot Cell.

[54] Heilmann C, Schneider S, Barker K, Rogers P, Morschhauser J (2010). An A643T mutation in the transcription factor Upc2p causes constitutive ERG11 upregulation and increased fluconazole resistance in Candida albicans.. Antimicrob Agents Chemother.

[55] Chamilos G, Nobile CJ, Bruno VM, Lewis RE, Mitchell AP (2009). Candida albicans Cas5, a regulator of cell wall integrity, is required for virulence in murine and toll mutant fly models.. J Infect Dis.

[56] Brown V, Sexton J, Johnston M (2006). A glucose sensor in Candida albicans.. Eukaryot Cell.

[57] Uppuluri P, Chaffin W (2007). Defining Candida albicans stationary phase by cellular and DNA replication, gene expression and regulation.. Mol Microbiol.

[58] Lan C, Rodarte G, Murillo L, Jones T, Davis R (2004). Regulatory networks affected by iron availability in Candida albicans.. Mol Microbiol.

[59] Sudbery P, Gow N, Berman J (2004). The distinct morphogenic states of Candida albicans.. Trends Microbiol.

[60] Chen C, Pande K, French SD, Tuch BB, Noble SM (2011). An Iron Homeostasis Regulatory Circuit with Reciprocal Roles in Candida albicans Commensalism and Pathogenesis.. Cell Host Microbes.

[61] Liu TT, Znaidi S, Barker KS, Xu L, Homayouni R (2007). Genome-Wide Expression and Location Analyses of the Candida albicans Tac1p Regulon.. Eukaryot Cell.

[62] Brun S, Aubry C, Lima O, Filmon R, Bergès T (2003). Relationships between respiration and susceptibility to azole antifungals in Candida glabrata.. Antimicrob Agents Chemother.

[63] Lupetti A, Paulusma-Annema A, Welling MM, Dogterom-Ballering H, Brouwer CPJM (2003). Synergistic activity of the N-terminal peptide of human lactoferrin and fluconazole against Candida species.. Antimicrobial Agents Chemother.

[64] Brun S, Dalle F, Saulnier P, Renier G, Bonnin A (2005). Biological consequences of petite mutations in Candida glabrata.. J Antimicrob Chemother.

[65] Zucchi PC, Davis TR, Kumamoto CA (2010). A Candida albicans cell wall-linked protein promotes invasive filamentation into semi-solid medium.. Mol Microbiol.

[66] Mitchell AP (1998). Dimorphism and virulence in Candida albicans.. Curr Opin Microbiol.

[67] Sexton JA, Brown V, Johnston M (2007). Regulation of sugar transport and metabolism by the Candida albicans Rgt1 transcriptional repressor.. Yeast.

[68] Chiranand W, McLeod I, Zhou H, Lynn J, Vega L (2008). CTA4 transcription factor mediates induction of nitrosative stress response in Candida albicans.. Eukaryot Cell.

[69] Oh J, Fung E, Schlecht U, Davis RW, Giaever G (2010). Gene annotation and drug target discovery in Candida albicans with a tagged transposon mutant collection.. PLoS Pathog.

[70] MacPherson S, Akache B, Weber S, De Deken X, Raymond M (2005). Candida albicans zinc cluster protein Upc2p confers resistance to antifungal drugs and is an activator of ergosterol biosynthetic genes.. Antimicrob Agents Chemother.

[71] Ondeyka J, Harris G, Zink D, Basilio A, Vicente F (2009). Isolation, structure elucidation, and biological activity of virgineone from Lachnum Wirgineum using the genome-wide Candida albicans fitness test.. J Nat Prod.

